# Renal resistive index: a new reversible tool for the early diagnosis and evaluation of organ perfusion in critically ill patients: a case report

**DOI:** 10.1186/s13089-019-0138-3

**Published:** 2019-10-10

**Authors:** Antonio Anile, Silvia Ferrario, Lorena Campanello, Maria Antonietta Orban, Giacomo Castiglione

**Affiliations:** 1 Intensive Care Unit, Ospedale Vittorio Emanuele, AOU Policlinico–Vittorio Emanuele, Catania, Italy; 2 Section of Nephrology, Department of Clinical and Experimental Medicine, Policlinico Universitario, University of Catania, Catania, Italy; 3 School of Anaesthesia and Intensive Care, University Hospital “G. Rodolico”, University of Catania, Catania, Italy

**Keywords:** Renal Doppler Resistive Index (RRI), Organ perfusion, Fluid challenge

## Abstract

**Background:**

We reported a case of early detection of peripheral hypoperfusion trough the evaluation of a new index in intensive care: Renal Doppler Resistive Index (RRI).

**Case presentation:**

We admitted a 76-year-old man who underwent ileostomy and hernioplasty because of an intestinal occlusion due to obstructive strangulated right inguinal hernia. The post-operative period was characterised by hemodynamic instability and he needed an invasive hemodynamic monitoring, administration of vasopressors and continuous renal replacement therapy (CRRT). Then, hemodynamic stability was obtained and vasopressors interrupted. RRI was lower than 0.7. In the eleventh post-operative day, despite stable macrocirculatory parameters, we found increased values of RRI. An abdomen ultrasound first and then a CT scan revealed the presence of bleeding from the previous ileostomy. Hence, the patient immediately underwent another surgical operation.

**Conclusions:**

RRI modification appears to be more precocious than any other hemodynamic, microcirculatory and metabolic parameter routinely used. RRI has been widely used to assess renal function in critically ill patients; now, we presume that RRI could represent a common and useful tool to manage target therapy in critical condition.

## Background

The macrohemodynamics targets, such as blood mean arterial pressure (MAP), cardiac output (CO), peripheral resistance, etc., have been used to drive goal-directed therapy in case of circulatory shock. However, it has been reported that conditions of microcirculatory disfunction could persist and evolve initially in hypoperfusion and then in cryptic shock [1] in spite of improvement or stability of macrocirculatory parameters. In cases of circulatory shock, monitoring microcirculatory parameters could be the most important endopoint to optimize the treatment, since microcirculatory disfunction could persist despite the improvement of macrocirculatory parameters [2]. Routine microcirculatory monitoring parameters, such as venous oxygen saturation (SvO2) and venous central oxygen saturation (SvcO2), remain fundamental but have several limits to consider [3]. SvO2 measurement implicates a pulmonary artery catheter. Collecting a sample of blood from superior caval vein allows to obtain a value of SvcO2 that reflects the O2 saturation of the superior vascular tree, that is slightly different as compared to O2 saturation of inferior vascular tree (as the abdominal vascular district) [1, 4]. Indeed, usually oxygen consumption (VO2) is independent of oxygen delivery (DO2) until tissues can satisfy metabolic requirement increasing oxygen extraction when DO2 decreases. This mechanism has an intrinsic limit: beyond critic DO2, the compensatory increasing of O2 extraction run out and VO2 become dependent from DO2.

Bloos et al. demonstrated that in patients with protract cardiac arrest, in case of microcirculatory artero-venous shunt or cellular apoptosis due to severe tissue damage, SvO2 and SvcO2 could show normal or increased values in spite of dangerous tissue hypoxia [3]. Consequently, tissue hypoxia develops and increased serum lactate and lactic acidosis may be observed [5–7].

Doppler-based renal resistive index measurement is a rapid and non-invasive tool that may be useful to detect tissue hypoperfusion and oxygenation, and to measure resistance to arterial blood flow in renal vessels in intensive care unit (ICU) patients [8, 9]. Sampling renal interlobar or arcuate arteries with pulsed wave Doppler ultrasound allows to obtain a “low resistance” profile typical of the downstream territories with high resting perfusion. Doppler waveform recorded in those territories shows a steep systolic rise that is the “early systolic peak”, followed by a decreasing component representing the diastolic flow.

RRI can be calculated as (peak systolic velocity − end diastolic velocity)/peak systolic velocity [10].

Evidence of a direct correlation between RRI and cardiovascular damages is more and more frequently reported; therefore, RRI has been proposed as a new tool in ICU patients monitoring [11, 12].

In normal conditions, renal artery blood flow occurs during both systolic and diastolic phase. On the contrary, in several pathologic conditions (such as shock, systemic inflammation, obstruction, etc.), renal arterial blood flow decreases and becomes even reverted during the diastolic phase consequently provoking an increase of RRI [8–12].

Nevertheless, RRI is the result of complex, and often not fully understood, interactions between arterial characteristics and systemic hemodynamic factors; in fact, many systemic parameters correlate with these ultrasonographic (US) measures. RRI does not always selectively indicate organ damage, but under particular conditions, it reflects systemic vascular disease. Increased values are associated with extrarenal factors such as age, vascular disease, pulse pressure, systemic vascular compliance, heart rate and cardiac function and with renal factor as end-stage renal disease and renal capillary wedge pressure. Thus, the hemodynamic factors involved have to be considered to understand RRI clinical meaning. Ageing provokes a progressive rigidity of the aorta with a great increase in the arterial pulse pressure. Pulse pressure is connected with cardiac function and systemic arterial compliance affecting the value of peak systolic velocity. The vascular compliance of large arteries determines the blood pressure pulsatility; consequently, in the condition of reduced systemic compliance, RRI results are strongly modified. The cardiac function, with its components such as heart rate and left ventricular outflow, can also strongly affect RRI.

Moreover, RRI is considered as a marker of progression of renal damage and an indicator of irreversible damage in chronic kidney failure.

Renal capillary wedge pressure develops from the combination of renal interstitial and venous pressure; therefore, conditions of systemic venous congestion could increase renal capillary wedge pressure with a subsequent elevation of RRI indexes [13].

Due to these confounding factors, as it has been previously suggested, it might be important to compare RRI with Splenic Doppler Resistive Index (SRI) [14]. Corradi et al. showed that the evaluation of SRI allows the early detection of occult hemorrhagic shock and persistent occult hypoperfusion after polytrauma in adult patients [15].

Since SRI variations after fluid administration could be an effective tool to monitor systemic hemodynamic, also SRI may represent a useful method in the detection of organ perfusion [16].

## Case report

A 76-year-old man was admitted to our hospital for abdominal pain and biliary vomiting. He had a past medical history of Alzheimer disease, chronic obstructive pulmonary disease and myelodysplastic syndrome recently treated with blood transfusion. He was diagnosed for intestinal occlusion due to obstructive right inguinal hernia and he was submitted to a surgical resection of an ischemic intestinal loop, ileostomy and hernioplasty. Due to hemodynamic instability, he was transferred to our ICU.

The post-operative period upon admission was characterised by hypotension non-responsive to fluid challenge and vasoplegia that required the administration of dopamine at the dosage of 6 mcg/kg/min to obtain hemodynamic stability. To achieve a better hemodynamic evaluation, an arterial catheter was inserted into femoral artery and a central venous catheter was inserted into internal jugular vein to monitor hemodynamic status using transpulmonary thermodilution (EV1000 system: Edwards Lifesciences, Irvine, CA). Cardiac index (CI) and mean arterial pressure were maintained > 4 l/min/m^2^ and 65–75 mmHg, respectively.

We recorded values of systemic vascular resistance index (SVRI) between 1000 and 1500 dyn s/cm^5^ m^2^. Administration of vasopressors was managed by hemodynamic monitoring with EV1000 (norepinephrine until 0.4 mcg/kg/min). During the first 24 h, a reduction of urine output was noted; hence, a fluid load was performed in association with high doses of diuretics. No response was obtained and we started calcium-citrate continuous venous–venous haemodialysis (calcium-citrate CVVHD Multifiltrate Fresenius System) with blood flow 100 ml/min, ultrafiltration 100 ml/h and dialysate flow 2000 ml/h. In the fourth postoperative day, we observed a recovery of renal function and we could suspend CVVHD.

**Fig. 1:**
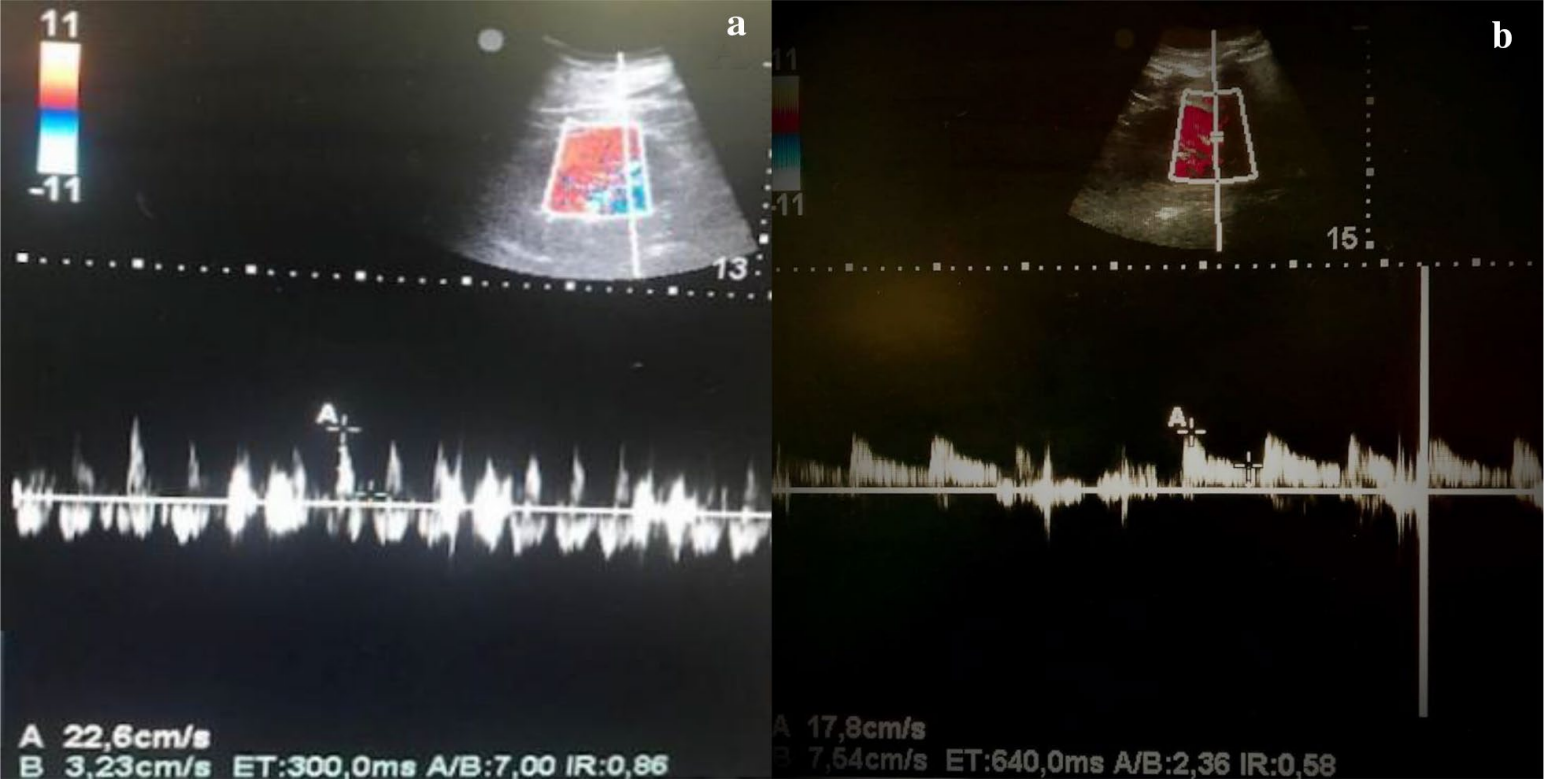
Renal Resistive Index. Pre- (**a**) and post- (**b**) surgical intervention of re-ileostomy

We monitored haemoglobin (Hb) level and we had to administrate blood transfusion to keep values of Hb > of 7.5 mg/dl. We also measured RRI in the first post-operative day and then every 48 h and we found values lower than 0.7 in each situation. Serum lactate constantly remained lower than 1.5 mEq/l. During the following days, the hemodynamic parameters recorded by EV1000 allowed to reduce vasopressors dosage until interruption of dopamine and decrease of norepinephrine to 0.08 mcg/kg/min. In the eleventh post-operative day, we performed another renal Doppler ultrasound to evaluate organ perfusion because of a condition of slightly tense abdomen and the need to increase norepinephrine dose. Despite stable macrocirculatory parameters recorded by EV1000, acceptable arterial blood gas analyses, normal level of SvcO2 (Table 1), we found increased values of RRI (0.86) (Fig. 1). Hence, we performed an abdomen Focused assessment with sonography for trauma (FAST) that allowed us to detect free intraabdominal fluid, and then we requested a CT scan that revealed the presence of bleeding from the previous ileostomy. We also measured the intra-abdominal pressure (IAP) that was 19 mmHg. Immediately afterwards, laboratory tests detected anaemia that required blood transfusion. The patient immediately underwent another surgical operation of blood evacuation (above 1000 ml), resection of the ileal loop responsible for the bleeding and new ileostomy was performed. After the operation, we measured IAP lesser of 11 mmHg. The hemodynamic monitoring with EV1000, arterial and venous blood gas analysis (Table 1) overlapped with the ones performed before the surgical operation. Despite the evidence of active bleeding from the previous ileostomy, detected with CT scan, the macroemodinamic parameters routinely used were still in normal range and weren't able to reveal hypoperfusion and haemodynamic instability. RRI instead showed an improvement of diastolic (perfusional) phase decreasing to normal values (0.58) (Fig. 1) and demonstrating the enhancement of organ perfusion.

**Table 1:** Macrocirculatory parameters from pre- (T0) and post-surgical (T1) intervention of re-ileostomy

	T0	T1
Lac	1	1.3
SvcO2	57	75
SVV %	4	13
SVRI	1018	1003
CI	4.6	5.3

*LAC* lactate (mmol/L), *SvcO2* venous central oxygen saturation (%), *SVV%* stroke volume variation (%), *SVRI* systemic vascular resistance index (dyn s/cm^5^ m^2^), *CI* cardiac index (L/min/m^2^)

## Discussion

It must be considered that the evaluation of RRI and SRI has limitations. First, all members of the medical équipe have to be able to evaluate these two ultrasonographic measures to establish a protocol of hemodynamic monitoring. Secondly, measuring RRI and SRI in not cooperative patients is not so easy. Thirdly, as mentioned above, RRI is influenced by many systemic extra-renal and renal factors that could conceal modifications caused by changes in organ perfusion.

Nevertheless, what we observed in our case suggests that RRI is an early and sensitive index of insufficient oxygen delivery and tissue hypoperfusion, probably reflecting vascular regulation even in initial phases of hypoxia. In addition, after the restoration of a sufficient microcirculatory blood flow and organ perfusion, the decrease in normal values shows the early reversibility of this index. The notion that shock or haemodynamic instability is a condition that does not imply necessarily a state of hypotension has been largely confirmed [17, 18]. Corradi et al reported that in haemodynamically stable multiple trauma patients, increased values of RRI could reliably predict a state of hidden or early haemorrhagic shock. Furthermore, in their study, serum lactate and Hb detected during the event were in normal ranges, showing that RRI is completely independent of MAP, CO and Hb [17, 18]. Moreover it is reported that BP and CO could not be considered unconditionally as markers of a shock developing [17]. Maynard et al. showed that peripheral hypoperfusion and “core organs” (splanchnic and mesenteric) hypoperfusion was an effective predictor of outcome other than regional perfusion [4]. Acting as functional supply reservoir, “core organs” protect themselves from hypotension until the insufficient DO2 determines cellular apoptosis and organ failure [2] becoming a multi-organ dysfunction syndrome (MODS) etiopathogenetic factor [1], as it has been demonstrated in animal and human models. A likely explanation could be found in the high sensitivity of kidney to ischemic damage owing to its complex microvascular structure along with its high metabolic needs [10]. Deruddre et al. showed that measurement of RRI could represent a new tool to identify the optimal individual MAP to sustain renal blood flow and could help managing norepinephrine titration during septic shock treatment [19]. More interesting is the early modification of RRI compared to SvcO2 which could be explained with a delay in SvcO2 increment hidden by a temporary increase of O2 extraction (up to a given limit) [3] that cannot be otherwise detectable or predictable by any other parameter.

In the early phase of shock in our patient, the routine hemodynamic parameters that conventionally indicate a state of hypoperfusion appeared to be unchanged; whereas RRI was significantly sensitive to hypoperfusion caused by IAP increment, that commonly determines renal injury and heart failure [20, 21].

To be also noted is the early reversibility of RRI, while the other haemodynamic monitored parameters (CO, SVV, MAP, blood gas analysis, SvcO2, Lactate and urine output) did not show perioperative modifications. To our knowledge, the only demonstration of a quantitative reversibility of intrarenal perfusion response was reported in animal models [22]. A meta-analysis recently published with the purpose to investigate the role of RRI as diagnostic tool showed that RRI may be considered as a predictor of persistent acute kidney injury (AKI) and its reversibility in critically ill patients [23].

If our observations were supported by large clinical trials, RRI could be considered as an early and reversible marker of organ perfusion that could be employed along with the other haemodynamic parameters routinely used.

## Data Availability

Not applicable.

## Abbreviations

RRI: Renal Doppler Resistive Index
CRRT: continuous renal replacement therapy
MAP: mean arterial pressure
CO: cardiac output
SvO2: venous oxygen saturation
SvcO2: venous central oxygen saturation
VO2: oxygen consumption
DO2: oxygen delivery
ICU: intensive care unit
CI: cardiac index
SVRI: systemic vascular resistance index
calcium-citrate CVVHD: calcium-citrate continuous venous–venous haemodialysis
Hb: haemoglobin
FAST: focused assessment with sonography for trauma
IAP: intra-abdominal pressure
AKI: acute kidney injury
SRI: Splenic Doppler Resistive Index
